# A simple and rapid method for detection of *Trypanosoma evansi *in the dromedary camel using a nested polymerase chain reaction

**DOI:** 10.1186/1475-9292-5-2

**Published:** 2006-05-20

**Authors:** Imadeldin E Aradaib, Ali A Majid

**Affiliations:** 1 Molecular Biology Research Unit, National Ribat University, Khartoum, Sudan; 2 Molecular Biology laboratory (MBL), Department of Medicine, Pharmacology and Toxicology, Faculty of Veterinary Medicine, University of Khartoum, P. O. Box 32, Khartoum North, Sudan; 3 National Council for Research, Khartoum, Republic of Sudan

## Abstract

A nested polymerase chain reaction (nPCR)-based assay, was developed and evaluated for rapid detection of *Trypanosoma evansi *in experimentally infected mice and naturally infected camels (*Camelus dromedarius*). Four oligonucleotide primers (TE1, TE2, TE3 and TE4), selected from nuclear repetitive gene of *T. evansi*, were designed and used for PCR amplifications. The first amplification, using a pair of outer primers TE1 and TE2, produced a 821-bp primary PCR product from *T. evansi *DNA. The second amplification, using nested (internal) pair of primers TE3 and TE4, produced a 270-bp PCR product. *T. evansi *DNAs extracted from blood samples of experimentally infected mice and naturally infected Sudanese breed of dromedary camels were detected by this nested PCR-based assay. The nested primers TE3 and TE4 increased the sensitivity of the PCR assay and as little as 10 fg of *T. evansi *DNA (equivalent to a single copy of the putative gene of the parasite) was amplified and visualized onto ethidium bromide-stained agarose gels.

Amplification products were not detected when the PCR-based assay was applied to DNA from other blood parasites including *Thieleria annulata, Babesia bigemina *or nucleic acid free samples. Application of this nPCR-based assay to clinical samples resulted in direct detection of *T. evansi *from a variety of tissue samples collected from experimentally infected mice and blood from naturally infected camels. The described nPCR-based assay provides a valuable tool to study the epidemiology of *T. evansi *infection in camels and other susceptible animal populations.

## Background

*Trypanosoma evansi *(*T. evansi*), the cause of trypanosomiasis (Surra), constitutes one of the major veterinary problems worldwide. The disease causes significant morbidity and mortality in camels in the Sudan, which has a population of over 3 million camels [1]. trypanosomiasis in camels occurs both in chronic and acute forms [2]. The chronic form of the disease is most common and is likely to be associated with secondary infections due to immunosuppression [3]. Clinical signs and pathological lesions caused by *T. evansi *in camels are unreliable for definitive diagnosis [4]. In addition, detection of parasites in the blood is difficult because parasitaemia is intermittent [5]. Serological tests have been developed and evaluated for diagnosis of trypanosomiasis in camels. They include card agglutination test and enzyme-linked immunosorbent assay (Ab-ELISA) [6-8]. In general serological techniques are useful for detection of a past infection but not for detection of an active infection with *T. evansi*. To address these problems, nucleic acid hybridization probes have been developed and evaluated for detection of *T. evansi*. However, the concentration of *T. evansi *DNA in suspected samples may be below the detection limit of the hybridization assay and hence positive cases are likely to be missed [9]. It is, therefore, becoming increasingly obvious that the development of molecular diagnostic techniques for rapid, sensitive and specific detection of *T. evansi *would be advantageous in a variety of circumstances including clinical disease investigation and epidemiologic surveys for the disease as well as in control programs. Recently, the polymerase chain reaction (PCR) has been widely used as highly sensitive and specific assay for detection of trypanosomes. Primers targeting subgroup Trypanozoon were previously described [10,11]. In addition, nuclear repetitive *T. evansi *specific PCR assays have also been developed [10-12]. The PCR has been used in surveys to determine the prevalence of *T. evansi *in camels from different regions in Kenya [3] and in buffaloes in Vietnam (Holland et al., 2001) and *T. equiperdum *in Mongolian horses [13]. However, PCR has not been systematically used for the detection of *T. evansi *in camels in Sudan. In this study, we described a simple, rapid, sensitive and specific assay for detection of *T. evansi *in Sudanese breed of dromedary camels (*Camelus dromedarius*) using nPCR amplification.

## Results

**Figure 1:**
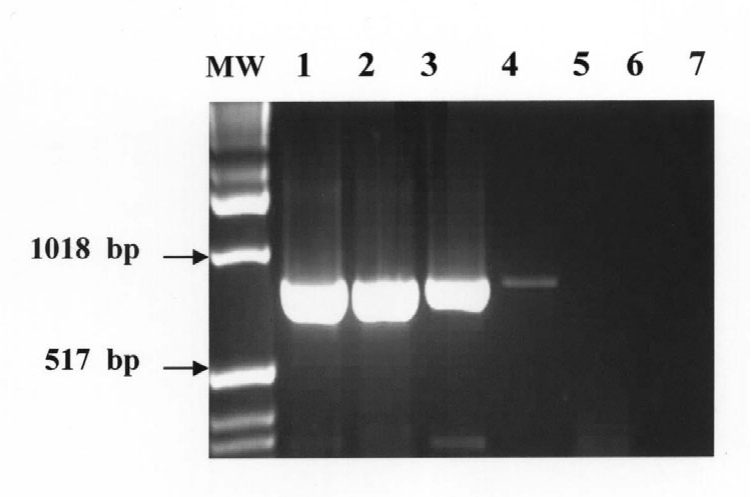
**Visualization of the primary 810-bp PCR product onto ethidium-bromide-stained agarose gel from 10 pg of DNA of *T. evansi***. Lane MW: molecular weight marker (1 Kb ladder); Lane 1–7: *T. evansi *DNA at a concentration of 10 ng, 1.0 ng, 100 pg, 10 pg, 1 pg, 100 fg, 10 fg, respectively.

**Figure 2:**
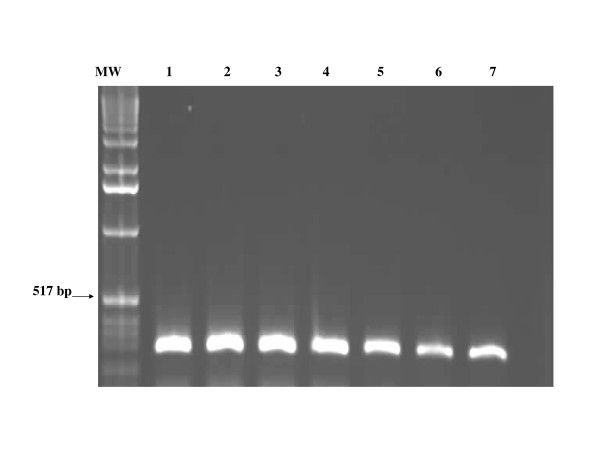
**Amplifcation of the nested 270-bp specific-PCR product on ethidium-bromide-stained agarose gel from 10 fg of *T. evansi *DNA**. Lane MW: molecular weight marker (1 Kb ladder); Lane 1–7: *T. evansi *DNA at a concentration of 10 ng, 1.0 ng, 100 pg, 10 pg, 1 pg, 100 fg, 10 fg, respectively.

The nPCR-based assay afforded sensitive and specific detection of all *T. evansi *strains obtained from experimentally infected mice and naturally infected camels. The outer pair of primers TE1 and TE2 produced a primary 810-bp PCR product from *T. evansi DNA*. The primary 810-bp PCR product was visualized on ethidium bromide-stained gel from ≥ 10 pg of *T evansi *DNA. (Figure 1). Using the nested primers (T3 and T4), the PCR assay resulted in amplification of a 270 bp PCR product from the primary PCR product. The nested 270 bp PCR product was detected from as little as as 10 fg of *T. evansi *DNA (equivalent to a single copy of nuclear repetitive gene of *T. evansi*). The nested amplification increased the sensitivity of the PCR assay and confirmed the identity of the nucleotide sequences of the primary amplified PCR product (Figure 2).

**Figure 3:**
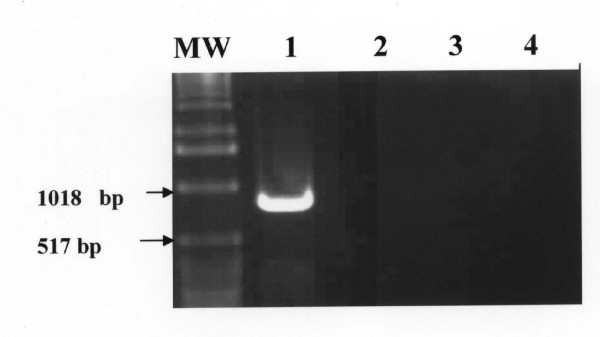
**Specificity of the polymerase chain reaction for *T. evansi*. The 810-bp PCR amplification product was not detected from 1.0 ng of DNA extracted from Babesia and Theileria spp**. Lane MW: molecular weight marker (1 Kb ladder); Lane 1: 1.0 ng of *T. evansi *DN (positive control), Lane 2: 1.0 pg DNA of *Theileria anulata*, Lane 3: 1.0 pg DNA of *Babesia bigemina*, Lane 4: nucleic acid free sample (negative control).

**Figure 4:**
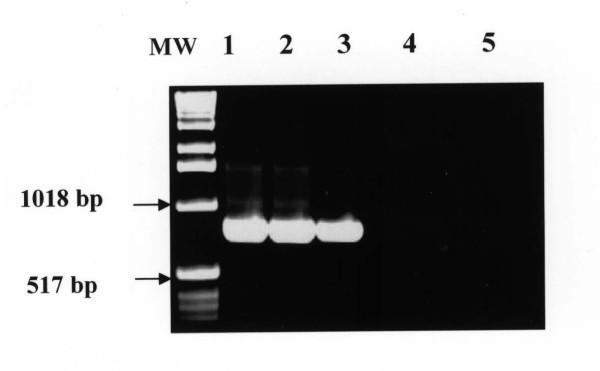
**Detection of the 810-bp *T. evansi *PCR product from tissue samples collected from experimentally infected mice**. Lane MW: molecular weight marker (1 Kb ladder); Lane 1: blood samples, Lane 2: spleen; Lane 3: liver; Lane: blood from non infected mouse, Lane 5: blood from non infected camel (negative controls).

**Figure 5:**
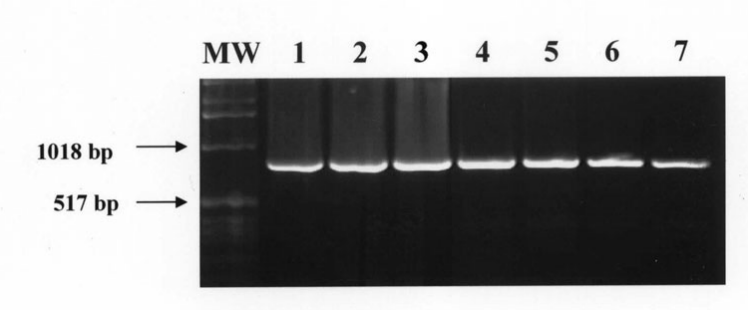
**Detection of the 810-bp *T. evansi *PCR product from blood samples collected from naturally infected camels**. Lane MW: molecular weight marker (1 Kb ladder); Lane 1–7: blood samples from naturally infected camels.

**Figure 6:**
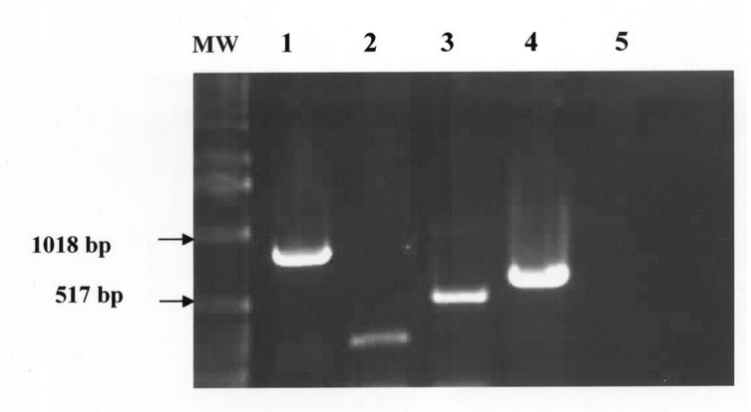
**Detection of *T. evansi *DNA by PCR using four different primers**. **Lane MW: **molecular weight marker (1 Kb ladder); Lane 1: amplification of the primary 810 bp-PCR product using the outer pair of primers (TE1 and TE2); Lane 2: amplification of the nested 270-bp PCR product using the internal pair of primers (TE3 and TE4); Lane 3: amplification of the semi-nested 490-bp PCR product using the pair of primers semi-nested 1 (TE1 and TE4); Lane 4: amplification of the semi-nested 590-bp PCR product using the pair of primers semi-nested 2 (TE3 and TE2). Lane 5: primers-free PCR amplification (negative control).

The amount of 1.0 ng DNA from other blood parasites including *Thieleria annulata, Babesia bigemina*; nucleic acid-free samples, blood and tissue samples from uninfected mice and camels failed to produce the primary or the nested amplification products (Figure 3). The specific PCR products were detected directly from a variety of infected tissue samples including, unfractionated lysed blood, spleen, lung and liver homogenates from experimentally infected mice (Figure 4) and from blood of naturally infected camels (Figure 5). Using the pair of primers semi-nested 1 (TE1 and TE4), the PCR assay resulted in amplification of a 490-bp PCR product. Likewise, the use of pair of primers semi-nested 2 (TE3 and TE2) produced a 590-bp PCR product (figure 6).

## Discussion

The economic importance of *T. evansi *infection is mainly attributed to clinical disease in camels in many parts of the world [14-18]. PCR has been used successfully in detecting infection with *T. evansi *in buffaloes [12,19], horses [13] and in camels [11]. So far, there is no comprehensive data on the use of PCR for detecting infection in Sudanese breed of dromedary camels (*Camelus dromedarius*). In the absence of other diagnostic techniques, the camel owners and ethno-veterinarians rely on the smell of urine for diagnosis of *T. evansi *infections. The characteristic smell of urine is due to ketones produced after breakdown of amino acids by the parasite in infected animals [20].

Conventional parasitological techniques will remain important for understanding the biology, ecology and molecular epidemiology of different strains of the parasites. The described nPCR assay provides simple, rapid, sensitive and specific method for detection of *T. evansi *in naturally infected camels (*Camelus dromedarius*) and can be recommended for inclusion in survey and control programmes. The nPCR-based assay using primers derived from nuclear repetitive gene of *T. evansi *reproducibly and specifically detected all strains of the parasite used in this study. Selection of these universal primers was based on the observation that the nuclear repetitive gene has highly conserved nucleotide sequences [11]. The nPCR assay was a simple procedure that efficiently detected all *T. evansi *strains under the stringency condition used in this study. The laboratory detection limit indicated that the nPCR protocol was capable of detecting the amount of 10 fg of total *T. evansi *genomic DNA.

The specificity studies indicated that the primary specific 821-bp and the nested 270-bp PCR products were not amplified even from concentrations of 10 ng of DNA from other blood parasite including *Thieleria annulata, Babesia bigemina *and blood cells from non infected mice and camels under the same stringency condition described in this study. Based on these sensitivity and specificity results of this study, the described nPCR should be considered as the most highly sensitive and specific assay compared to the previously described PCR-based detection assays [10,12,13,19]. The second amplification step using the nested primers TE3 and TE4 is necessary to confirm the specificity of the primary amplified product and to increase the sensitivity of the PCR-based assay by at least 1000 times particularly, when the concentration of the *T. evansi *DNA in the sample is less than 10 pg. The use of nested amplification for confirmatory diagnosis of *T. evansi *infection renders this PCR assay a rapid and an inexpensive assay. The nested PCR does not required hybridization assay and this removes the hazardous and cumbersome radioactive laboratory procedures of working with ^32^P or ^33^P in hybridization assays [23]. Successful amplification was also obtained from tissue samples from whole blood of experimentally infected mice, unlike other PCR assays where fractionation of blood leukocyte was necessary for detection of the parasite DNA [10]. Sample preparation and DNA extraction using QIAamp extraction kit was a simple procedure, which takes one hour. The thermal cycling profiles for production of the primary and the nPCR products were consistently 4 hours. Running of the agarose gel and electrophoresis usually takes one hour. Thus, confirmatory diagnosis of *T. evansi *infection using the described nPCR assay could be made within the same working day.

Cross contamination in nPCR amplification reactions is also a problem. The problem of contamination could be minimized by using nucleic acid-free aerosol resistant tips and separate rooms for DNA extraction and amplification. Negative and positive controls should be included in each PCR assay to maintain the higher limit of sensitivity and the lower limit of specificity. The rapidity, sensitivity and specificity of the nPCR assay would greatly facilitate rapid detection of early or chronic infection with *T. evansi *in camels and other susceptible animals. Application of this nested PCR on a practical scale to determine the prevalence of *T. evansi *in naturally infected camels in different states of Sudan is in progress.

## Conclusion

The described nPCR assay, using well characterized *T. evansi*-specific primers, provides a simple, rapid, sensitive and specific detection of *T. evansi *in naturally infected camels (*Camelus dromedarius*) and can be used as a valuable tool during epidemiological surveys and control programmes.

## Methods

### Collection of blood samples

Blood samples were collected in clean sterile vacutainers, containing ethylene diamine tetra acetic acid (EDTA), from experimentally infected mice and from naturally infected camels. The blood samples were used for extraction of total nucleic acids for use as target DNA for PCR amplification.

### Parasitological examination and mouse inoculation

Two capillary tubes were prepared from each heparinised blood sample and centrifuged in a microhaematocrit centrifuge for 5 min at 12,000 g. The capillary tubes were examined for the presence of motile trypanosomes using the microhaematocrit centrifugation technique. Four hundred microlitres of blood from infected camels were inoculated into Swiss-bred mice, which were screened for infection with *T. evansi *by wet smear examination of tail-tip blood every day for detection of parasitaemia.

### Extraction of DNA from blood samples

Whole blood was used for extraction of total genomic DNA using commercially available QIAamp blood kit (QIAGEN Inc. Chatsworth, CA, USA) according to the manufacturer's instructions. Briefly, 200 μl of whole blood, 20 μl of proteinase K stock solution, and 200 μl of lysing buffer were pipetted into 1.5 ml eppendorf tube and the mixture was incubated at 37°C for 30 minutes and then at 70°C for 10 minutes. 200 μl of absolute ethanol was added to the sample and the mixture was mixed by vortexing and spinning. The mixture was transferred to the QIAspin column, and placed in a clean 2 ml collection tube and centrifuged at 8000 g for 1 minute. The QIAspin column was washed twice using 500 μl of washing buffers W1 and W2, respectively. The QIAamp spin column was then placed in a clean 1.5 ml eppendorf tube and the DNA was eluted with 200 μl double distilled water preheated at 70°C. Maximum DNA yield was obtained by spinning at 12,000 g for 1 min. The DNA concentration was determined by spectrophotometer at 260 nm wave length. Five μl of the extracted DNA was used in the PCR amplification.

### Extraction of DNA from tissue samples

Five ml distilled water was added to 1 gm of each tissue sample collected from lungs, Liver and spleen of experimentally infected mice. The tissue sample was then mixed by homogenization. The homogenate was treated by freezing and thawing and finally centrifuge at 3000 RPM for 10 minutes. 200 μl of the supernatant was used for DNA extraction following the same procedure used for extraction of DNA from blood as described above.

### Primers selection

Primers (TE1 and TE2) were selected from a highly conserved region of the published sequence of nuclear repetitive gene of Indonesian strain of *T. evansi *[24]. This pair of primers was used for the synthesis of the primary PCR amplification product. TE1 included bases 91–110 of the positive sense strand of *T. evansi *putative gene (5)-AGG ACG CAG AAA TAG CAG TA-(3). TE2 included bases 881–900 of the complementary strand: (5)-ATT TAA TTG AGT GGC GTG AG-(3). The PCR using primer TE1 and TE2 will result in a 810-bp PCR product. For the nested amplification step, oligonucleotide primers (TE3 and TE4) were selected from the same published sequence cited above. TE3 consisted of bases 311–330 of the positive strand (5)-CTT TTA TAC GAG GAG AGG GA-(3). TE4 was designed from the complementary strand between bases 561–580 (5)-TAT GGG CGT GCA GAT TTC AC-(3). PCR amplification using TE3 and TE4 will result in a 270 bp PCR product, internal to the annealing sites of TE1 and TE2. Using the semi-nested pair of primers (TE1 and TE4), the PCR assay will result in amplification of a 490-bp semi-nested PCR product. The pair of semi-nested primers (TE3 and TE2) will be expected to produce a 590-bp semi-nested PCR product. Details for primers design including position of nucleotides, nucleotide sequences, and expected PCR products are shown in (Table 1).

**Table 1:** 

**Primer**	**Position**	**Nucleic acid sequence**	**PCR product**
External (outer primers)			
TE1	91–110	(5)-AGGACGCAGAAATAGCAGTA (3)	810-bp
TE2	881–900	(5)-ATTTAATTG AGTGGCGTGAG (3)	
Internal (nested Primers)			
TE3	311–330	5)-CTTTTATACGAGGAG GGGA (3)	270-bp
TE4	561–580	5)-TATGGGCGTGCAGATTTC AC (3)	
Semi-nested 1			
TE1	91–110	5)-AGGACGCAGAAATAGCAGTA (3)	490-bp
TE4	561–580	5)-TATGGGCGTGCAGATTTC AC (3)	
Semi-nested 2			
TE3	311–330	5)-CTTTTATACGAGGAG GGGA (3)	590-bp
TE2	881–900	5)-ATTTAATTG AGTGGCGTGAG (3)	

All primers were synthesized on a DNA synthesizer (Milliigen/Biosearch, a division of Millipore Burlington, MA) and purified using oligo-pak oligonucleotide purification columns (Glen Research Corporation, Sterling, VA.) as per manufacturer's instructions.

### Polymerase chain reaction (PCR)

A stock buffered solution containing 250 μl 10× PCR buffer, 100 μl of magnesium chloride, 12.5 μl of each dATP, dTTP, dGTP and dCTP was prepared in 1.5 ml eppendorf tube. The primers were used at a concentration of 20 pg/μl, and double distilled water was added to bring the volume of the stock buffer solution to 1.5 ml. Two μl of primers, 5.0 μl of the target DNA and 42 μl of the stock solution were added onto 0.5 ml PCR tubes and mixed by vortexing. One μl of Taq DNA polymerase (Perkin Elmer) was used at a concentration of 5.0 U/μl. All PCR amplification reactions were carried out in a final volume of 50 μl. The thermal cycling profiles were as follows: a 2 min initial incubation at 95°C, followed by 40 cycles of 95°C for 1 min, 55°C for 30 sec and 72°C for 45 sec, and a final incubation at 72° for 10 min. Thermal profiles were performed on a Techne PHC-2 thermal cycler (Techne, Princeton, NJ.). Following amplification, 15 μl from each PCR containing amplified product were loaded onto gels of 1.0% SeaKem agarose (FMC Bioproduct, Rockland ME) and electrophoresed. The gels were stained with ethidium bromide and *T. evansi *primary PCR products were easily identified following visualization under UV light.

### Nested Polymerase Chain Reaction (nPCR)

For the nested PCR amplification, 2.0 μl of the primary amplified product produced by TE1 and TE2 were transferred to 0.5 ml PCR tube containing (2 μl of nested primers and; 42 μl of stock PCR buffer and Taq DNA polymerase was used at a concentration of 5.0 U/μl. The nested pair of primers (TE3 and TE4) was expected to amplify a 270 bp PCR product, internal to the annealing sites of primers PSL1 and PSR2. All PCR amplifications were carried out in a final volume of 50 μl. The thermal cycling profiles were as follows: a 2 min incubation at 95°C, followed by 30 cycles of 94°C for 1 min, 55°C for 30 sec and 72°C for 45 sec, and a final incubation at 72°C for 10 min. Thermal profiles were performed on a Techne PHC-2 thermal cycler (Techne, Princeton, NJ.). Following amplification, 15 μl from each PCR containing amplified products were loaded onto gels of 1.5% SeaKem agarose (FMC Bioproduct, Rockland ME) and electrophoresed. The gels were stained with ethidium bromide and the nested PCR products were easily identified following visualization under UV light.

## Competing interests

The author(s) declare that they have no competing interests.

## Authors' contributions

AAM designed the study and prepared the manuscript. IEA performed the experimental work and optimized the nested polymerase chain reaction-based detection assay.
